# Radiosensitization of Normoxic and Hypoxic H1339 Lung Tumor Cells by Heat Shock Protein 90 Inhibition Is Independent of Hypoxia Inducible Factor-1α

**DOI:** 10.1371/journal.pone.0031110

**Published:** 2012-02-07

**Authors:** Daniela Schilling, Christine Bayer, Wei Li, Michael Molls, Peter Vaupel, Gabriele Multhoff

**Affiliations:** 1 Department of Radiation Oncology, Klinikum rechts der Isar, Technische Universität München, Munich, Germany; 2 Clinical Cooperation Group (CCG) “Innate Immunity in Tumor Biology”, Helmholtz Zentrum München, Munich, Germany; 3 Department of Dermatology, University of Southern California Keck School of Medicine, Los Angeles, California, United States of America; National Cancer Institute, United States of America

## Abstract

**Background:**

Ionizing irradiation is a commonly accepted treatment modality for lung cancer patients. However, the clinical outcome is hampered by normal tissue toxicity and tumor hypoxia. Since tumors often have higher levels of active heat shock protein 90 (Hsp90) than normal tissues, targeting of Hsp90 might provide a promising strategy to sensitize tumors towards irradiation. Hsp90 client proteins include oncogenic signaling proteins, cell cycle activators, growth factor receptors and hypoxia inducible factor-1α (HIF-1α). Overexpression of HIF-1α is assumed to promote malignant transformation and tumor progression and thus might reduce the accessibility to radiotherapy.

**Methodology/Principal Findings:**

Herein, we describe the effects of the novel Hsp90 inhibitor NVP-AUY922 and 17-allylamino-17-demethoxygeldanamycin (17-AAG), as a control, on HIF-1α levels and radiosensitivity of lung carcinoma cells under normoxic and hypoxic conditions. NVP-AUY922 exhibited a similar biological activity to that of 17-AAG, but at only 1/10 of the dose. As expected, both inhibitors reduced basal and hypoxia-induced HIF-1α levels in EPLC-272H lung carcinoma cells. However, despite a down-regulation of HIF-1α upon Hsp90 inhibition, sensitivity towards irradiation remained unaltered in EPLC-272H cells under normoxic and hypoxic conditions. In contrast, treatment of H1339 lung carcinoma cells with NVP-AUY922 and 17-AAG resulted in a significant up-regulation of their initially high HIF-1α levels and a concomitant increase in radiosensitivity.

**Conclusions/Significance:**

In summary, our data show a HIF-1α-independent radiosensitization of normoxic and hypoxic H1339 lung cancer cells by Hsp90 inhibition.

## Introduction

Lung cancer is one of the most frequent tumors worldwide and patients with locoregionally advanced tumor stages are frequently treated with radiochemotherapy. However, the efficacy of radiotherapy is limited by genetic and epigenetic diversity [1] and extrinsic factors such as hypoxia [2], [3]. The adaptation of tumor cells to hypoxia is primarily mediated by stabilization of two hypoxia inducible factor (HIF) complexes, HIF-1 and HIF-2 [4]. HIF-1 and HIF-2 are heterodimeric transcription factors composed of a constitutively expressed β-subunit (HIF-1β/ARNT) and a HIF-α subunit (HIF-1α, HIF-2α), which is regulated by tissue oxygen status. Under normoxia, hydroxylation of the HIF-α subunit by prolyl hydroxylase domain (PHD) proteins enables binding of the von Hippel-Lindau tumor suppressor protein (VHL) that rapidly targets the HIF-α subunit to ubiquitination and proteasomal degradation. Under hypoxia, the degradation pathway is suppressed and the HIF-α subunit accumulates, translocates into the nucleus and dimerizes with the HIF-β subunit. The HIF complex binds to the hypoxia responsive element (HRE) in the promoter region of oxygen-regulated genes and leads to their transcriptional activation. Although the majority of these genes are involved in the adaptation of tumor cells to hypoxic conditions (e.g., shift to glycolytic metabolism), a substantial proportion also contributes to tumor progression by inducing angiogenesis, local invasion and metastatic dissemination [2].

The HIF-α subunits have been shown to interact with the molecular chaperone heat shock protein 90 (Hsp90) [5]. Hsp90 is involved in the posttranslational folding and stabilization of more than 200 client proteins which are required for the activity of key regulators of cell signaling that promote tumor cell growth and radioresistance [6]. Hsp90 is frequently overexpressed in tumors and therefore, inhibition of Hsp90 has emerged as a potential drug target in cancer therapy. The first Hsp90 inhibitor that entered clinical trials up to phase III was 17-allylamino-17-demethoxygeldanamycin (17-AAG), a derivative of the natural antibiotic geldanamycin [6]. Despite its anti-tumor activity, 17-AAG is hampered by its poor water solubility, toxicity and limited absorption. The synthetic, isoxazole/ resorcinol-based Hsp90 inhibitor NVP-AUY922 is a second generation Hsp90 inhibitor which leads to tumor growth inhibition *in vitro* and regression of human tumor xenografts in mice [7]. Compared to 17-AAG, NVP-AUY922 exhibits a tighter binding to Hsp90 and is metabolically more stable [8]. Due to its improved pharmacokinetics and bioavailability, NVP-AUY922 is expected to be more effective than 17-AAG. Since Hsp90 interferes with a variety of pathways (including DNA repair [9]) which are known to protect tumor cells from irradiation-induced death [9], [10], Hsp90 inhibition is assumed to improve the outcome of radiotherapy.

Increased levels of HIF-1α or HIF-2α have been associated with resistance of tumors to irradiation [11], [12], although, the role of Hsp90 inhibitors in the regulation of HIF is not completely understood. Therefore, we have analyzed the effects of NVP-AUY922 and 17-AAG on the HIF-1α/HIF-2α expression in combination with radiosensitivity in lung cancer cell lines under normoxic and hypoxic conditions.

## Results

### Hsp90 inhibitors increase HIF-1α levels in H1339 lung cancer cells

**Figure 1:**
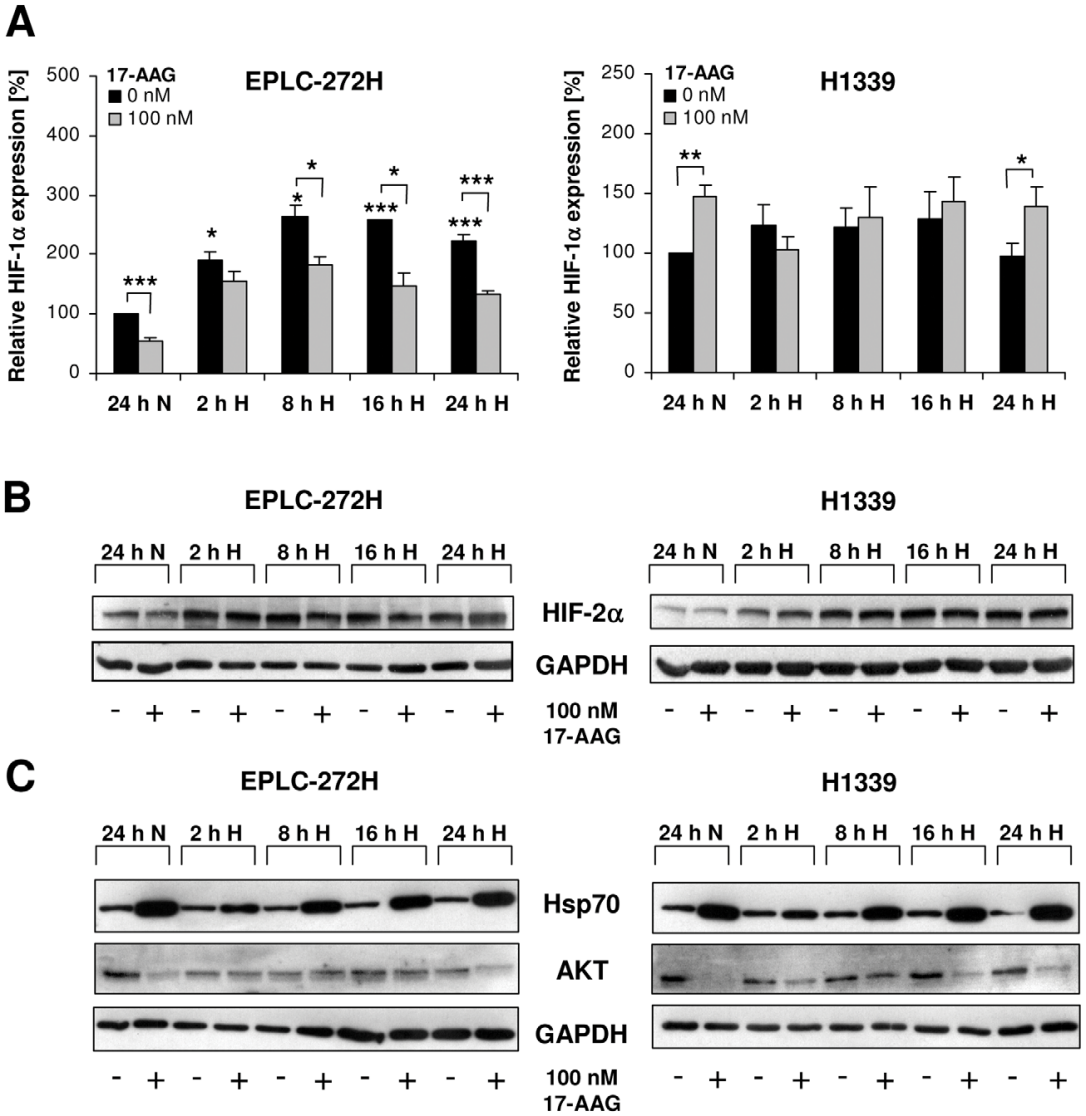
Time kinetics of HIF-1α, HIF-2α and Hsp70 levels after treatment with 17-AAG and exposure to hypoxia. (A) HIF-1α expression levels in EPLC-272H (left panel) and H1339 (right panel) cells treated with 17-AAG and subsequently (30 min later) exposed to normoxia for 24 h (24 h N) or hypoxia for 2 h (2 h H), 8 h (8 h H), 16 h (16 h H) and 24 h (24 h H) were determined by ELISA. Mean values ± SEM of at least three independent experiments are shown. *p≤0.05, **p≤0.01, ***p≤0.001. (B and C) Representative HIF-2α (B), Hsp70 and AKT (C) immunoblots of EPLC-272H and H1339 cells treated with 17-AAG and subsequently (30 min later) exposed to normoxia for 24 h (24 h N) or hypoxia for 2 h (2 h H), 8 h (8 h H), 16 h (16 h H) and 24 h (24 h H).

Since Hsp90 co-immunoprecipitates with HIF-α subunits [5], Hsp90 inhibition has gained attention in targeting hypoxic signaling. Herein, HIF-1α and HIF-2α protein levels were analyzed in EPLC-272H and H1339 lung cancer cells under normoxic ([O_2_] = 21%) and hypoxic ([O_2_] = 0.66%) conditions, in the presence and absence of two structurally distinct Hsp90 inhibitors, 17-AAG and NVP-AUY922. Under normoxia, EPLC-272H cells express low levels of HIF-1α (697±117 pg/mg protein) that are more than doubled following a 24 h hypoxia treatment (1574±286 pg/mg protein). In contrast, H1339 cells exhibit high basal HIF-1α levels already under normoxic conditions (1546±296 pg/mg protein) which were not further enhanced by hypoxia (1375±282 pg/mg protein). Kinetic studies revealed significantly increased HIF-1α levels from 2 to 24 h after hypoxia in EPLC-272H cells (Fig. 1A, black bars, left graph; *p≤0.05; ***p≤0.001), whereas the high basal HIF-1α levels remained unaffected in H1339 cells (Fig. 1A, black bars, right graph). As demonstrated previously, the inability of H1339 cells to up-regulate HIF-1α in response to hypoxia can neither be explained by varying cell densities, absence / presence of growth factors nor by reoxygenation effects [13]. In contrast to HIF-1α, HIF-2α was up-regulated upon hypoxic exposure in both tumor cell lines (Fig. 1B). In accordance with findings of other groups [14], G_1_-phase was up- and S-phase was down-regulated upon hypoxic exposure in H1339 cells (Fig. S1). Taken together, these data indicate functional hypoxic signaling in H1339 cells although HIF-1α expression was not affected.

**Figure 2:**
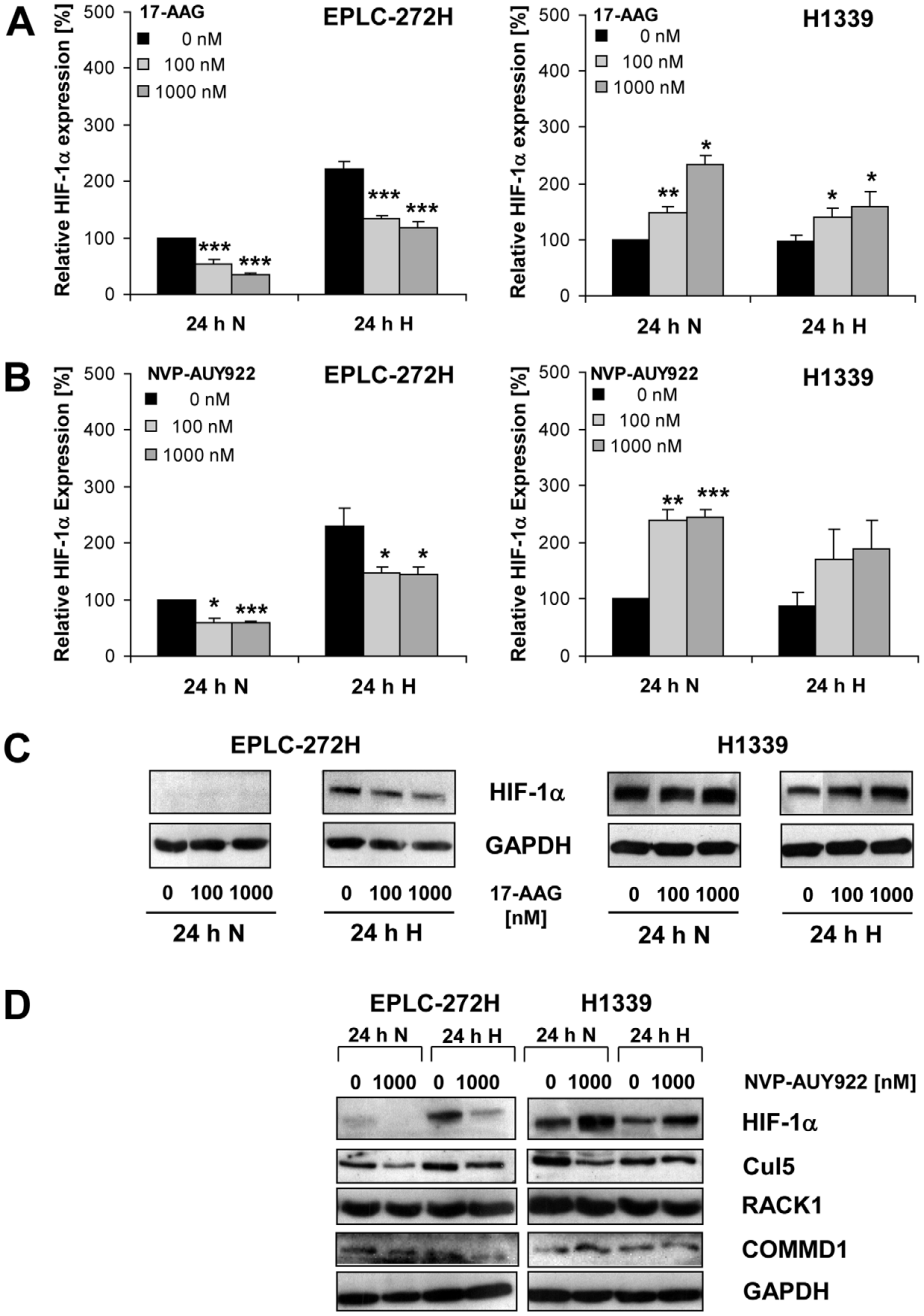
17-AAG and NVP-AUY922 enhance HIF-1α levels in H1339 lung cancer cells. (A and B) HIF-1α expression levels in EPLC-272H and H1339 cells treated with 0, 100 and 1000 nM 17-AAG (A) or with 0, 100 and 1000 nM NVP-AUY922 (B) and subsequently (30 min later) exposed to normoxia for 24 h (24 h N) or hypoxia for 24 h (24 h H) were determined by ELISA. Mean values ± SEM of at least three independent experiments are shown. *p≤0.05, **p≤0.01, ***p≤0.001. (C) Representative HIF-1α immunoblots of EPLC-272H and H1339 cells treated with 0, 100 and 1000 nM 17-AAG and subsequently (30 min later) exposed to normoxia for 24 h (24 h N) or to hypoxia for 24 h (24 h H). (D) Representative HIF-1α, Cul5, RACK1 and COMMD1 immunoblots of EPLC-272H and H1339 cells treated with 0 and 1000 nM NVP-AUY922 and subsequently (30 min later) exposed to normoxia for 24 h (24 h N) or to hypoxia for 24 h (24 h H).

As expected, Hsp90 inhibition caused a significant down-regulation of hypoxia-induced HIF-1α levels from 8 to 24 h after exposure to 17-AAG in EPLC-272H cells (Fig. 1A, grey bars, left graph; *p≤0.05, ***p≤0.001). In H1339 cells, however, the elevated basal HIF-1α levels were further up-regulated 24 h after treatment with 17-AAG under normoxic and hypoxic conditions (Fig. 1A, grey bars, right graph; *p≤0.05, **p≤0.01). Similar results were obtained by using the small molecule Hsp90 inhibitor NVP-AUY922 (Fig. 2B).

The administration of 17-AAG (100 nM; Fig. 1B) and NVP-AUY922 (data not shown) did not significantly affect the HIF-2α levels in both cell lines. These results suggest that stabilization of HIF-2α in the two cell lines investigated might be regulated by a mechanism which differs from that of HIF-1α.

The effectiveness of the Hsp90 inhibition by 17-AAG in EPLC-272H and H1339 tumor cells under normoxic and hypoxic conditions was proven by a strong induction of the synthesis of Hsp70 (Fig. 1C). It is well established that Hsp90 inhibitors stimulate the activity of heat shock factor 1 (HSF1), a transcription factor which is essential for the induction of the Hsp70 expression. Therefore, up-regulation of the cytosolic Hsp70 levels provides a surrogate marker for functional Hsp90 inhibition [15]. Hsp90 inhibition was also demonstrated by the down-regulation of the Hsp90 client protein AKT in both cell lines by a 24 h 17-AAG treatment (Fig. 1C).

Since most prominent differences with respect to HIF-1α were measured 24 h after 17-AAG treatment (Fig. 1A), further dose response analyses were performed at this time-point. Drug-induced toxicity could be excluded since a treatment of tumor cells with 100 and 1000 nM 17-AAG or NVP-AUY922 for 24 h did not reduce cell viability (Table S1).

HIF-1α levels were significantly down-regulated in normoxic and hypoxic EPLC-272H cells by 17-AAG (Fig. 2A, left panel) and NVP-AUY922 (Fig. 2B, left panel) at inhibitor concentrations of 100 and 1000 nM. In contrast, the same concentrations of 17-AAG (Fig. 2A, right panel) and NVP-AUY922 (Fig. 2B, right panel) induced an up-regulation of HIF-1α in normoxic and hypoxic H1339 cells. These results derived by ELISA were confirmed by Western blot analysis (Fig. 2C). Since a similar HIF-1α expression pattern was observed using two structurally distinct Hsp90 inhibitors, we conclude that HIF-1α regulation rather depends on the tumor cell type than on the chemical structure of the Hsp90 inhibitor.

In order to explain the differential regulation of HIF-1α after treatment with Hsp90 inhibitors in H1339 and EPLC-272H lung cancer cells, we investigated the expression of Cullin 5 E3 ubiquitin ligase (Cul5), receptor of activated protein C kinase (RACK1) and Copper Metabolism MURR1 Domain containing 1 protein (COMMD1) which have been described to regulate the degradation of HIF-1α upon Hsp90 inhibition [16]–[18]. As shown in Figure 2D, the lung carcinoma cell lines with differential HIF-1α expression did not differ in the expression pattern of these proteins.

### 17-AAG and NVP-AUY922 differentially affect HIF-1α target gene products in lung cancer cells

**Figure 3:**
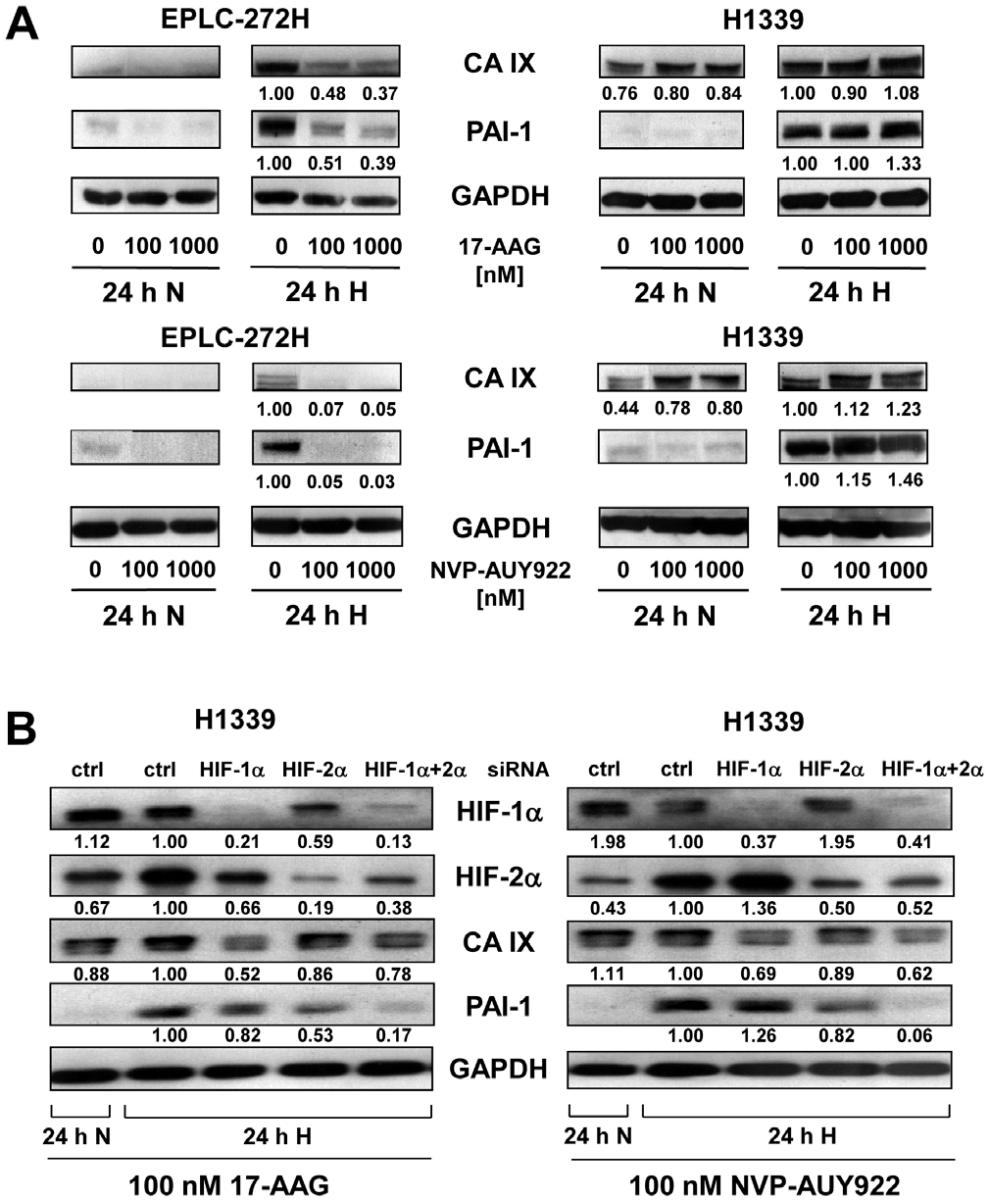
17-AAG- or NVP-AUY922- induced HIF-1α levels in H1339 lung cancer cells are functional. (A) Representative CA IX and PAI-1 immunoblots of EPLC-272H and H1339 cells treated with 0, 100 and 1000 nM 17-AAG or NVP-AUY922 and 30 min later exposed to normoxic (24 h N) or hypoxic (24 h H) conditions for 24 h. The protein bands were quantified by densitometry using ImageJ. Numbers under the lanes represent the expression of CA IX or PAI-1 relative to GAPDH. The value of 24 h hypoxia (24 h H) without drug treatment (0 nM) was set as 1 for each experiment. (B) Representative HIF-1α, HIF-2α, CA IX and PAI-1 immunoblots of H1339 cells transfected with a non-targeting (ctrl), HIF-1α, HIF-2α or both HIF-1α and HIF-2α siRNA. At 24 h after transfection, cells were treated with 100 nM 17-AAG or NVP-AUY922 and 30 min later exposed to normoxic (24 h N) or hypoxic (24 h H) conditions for 24 h. The protein bands were quantified by densitometry using ImageJ. Numbers under the lanes represent the expression of HIF-1α, HIF-2α, CA IX or PAI-1 relative to GAPDH. The value of 24 h hypoxia (24 h H) transfected with non-targeting siRNA (ctrl) was set as 1 for each experiment.

In order to determine how Hsp90 inhibition influences down-stream HIF target gene products, the expression levels of carbonic anhydrase IX (CA IX) and plasminogen activator inhibitor type-1 (PAI-1) were investigated following treatment with 17-AAG or NVP-AUY922. CA IX is known to be regulated by HIF-1α [19], whereas PAI-1 is described to be controlled by HIF-1α and/or HIF-2α [20]–[22]. Hypoxia causes a strong induction of CA IX and PAI-1 expression in EPLC-272H cells, which was down-regulated after addition of increasing concentrations of 17-AAG or NVP-AUY922 (Fig. 3A, left). In contrast to EPLC-272H cells, H1339 cells show similar CA IX levels under normoxia and hypoxia which are increased by 17-AAG or NVP-AUY922 (Fig. 3A, right). The same pattern was observed for HIF-1α levels (Fig. 2A–C) suggesting that Hsp90 inhibition modulates CA IX levels in H1339 cells via HIF-1α. PAI-1 expression of H1339 cells is induced by hypoxia and Hsp90 inhibitors (Fig. 3A, right). GAPDH was used as loading control since it has been shown to be a suitable housekeeping gene under hypoxic conditions (Fig. S2) [23].

### 17-AAG- and NVP-AUY922-induced HIF-1α is functional in H1339 lung cancer cells

The functionality of the increased HIF-1α proteins in H1339 cells upon Hsp90 inhibition was proven by measuring down-stream target gene products, such as CA IX and PAI-1 after HIF-1α knock-down. As shown in Fig. 3B, HIF-1α expression is specifically inhibited by HIF-1α siRNA, whereas the HIF-2α levels remained unaffected and vice versa. A mixture of HIF-1α and HIF-2α siRNA resulted in a reduction of both HIF-α subunits (Fig. 3B).

The expression of CA IX was specifically inhibited by transfection with HIF-1α but not HIF-2α siRNA (Fig. 3B). A simultaneous knock-down of HIF-1α and HIF-2α caused a decrease in CA IX expression which is comparable to the individual HIF-1α knock-down. In contrast to CA IX, PAI-1 expression was reduced by HIF-2α, but not by HIF-1α siRNA. Furthermore, sequencing of the HIF-1α cDNA of H1339 lung cancer cells revealed no changes to the published HIF-1α coding sequence (NCBI Reference Sequence: NM_001530.3). These data demonstrate that the Hsp90 inhibitor-induced HIF-1α levels were not mutated and functional in H1339 cells, since CA IX is down-regulated by a HIF-1α knock-down.

### Hsp90 inhibition enhances radiosensitivity of H1339 cells regardless of HIF-1α levels

**Figure 4:**
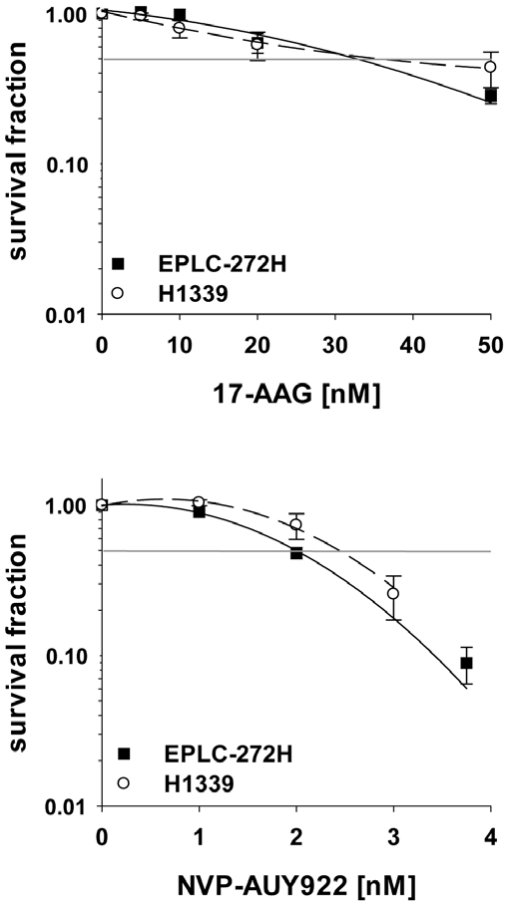
EPLC-272H and H1339 lung cancer cell lines show similar drug-sensitivity. EPLC-272H and H1339 cells were exposed to vehicle control (0 nM), 17-AAG (5–50 nM) or NVP-AUY922 (1–3.75 nM). Drugs were present until fixation of colonies. The survival fraction was calculated relative to the untreated control. Horizontal lines indicate 50% survival fraction. Mean values ± SEM of at least 4 independent experiments are shown.

Based on low basal levels of HIF-1α in EPLC-272H and high levels in H1339 tumor cells, a higher sensitivity towards Hsp90 inhibition was expected for EPLC-272H cells. However, the sensitivity towards drug alone was comparable for both tumor cell lines, whereby NVP-AUY922 (IC_50_: 2.5 nM for H1339; 2.0 nM for EPLC-272H) was 15-times more effective than 17-AAG (IC_50_: 30.8 nM for H1339; 30.0 nM for EPLC-272H) (Fig. 4). Despite varying HIF-1α levels, tumor cell lines EPLC-272H and H1339 also showed a comparable resistance towards irradiation (SF_2_: 0.64 for EPLC-272 and 0.76 for H1339). Although elevated HIF-1α levels were previously reported to be predictive for radioresistance [11] our findings show an increased radiosensitivity in tumors with high basal HIF-1α levels. This finding was confirmed in a larger panel of tumor cell lines derived from different entities [24].

**Figure 5:**
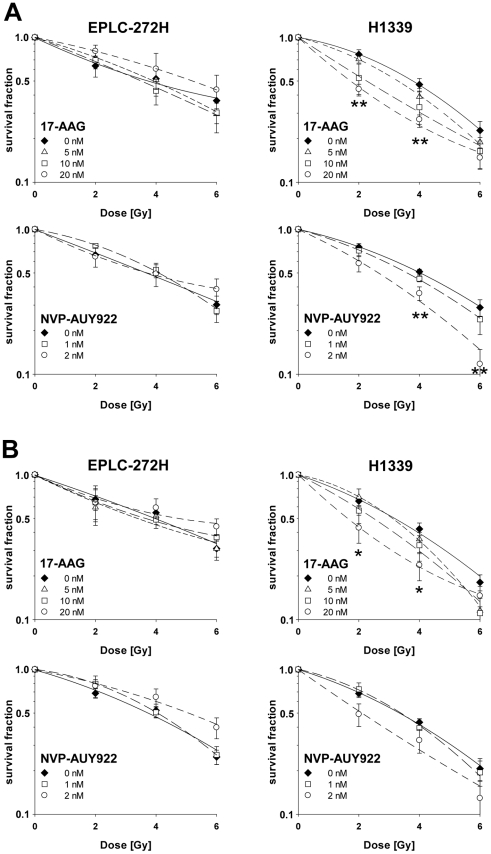
Hsp90 inhibitors increase radiosensitivity of H1339 lung cancer cells under normoxic and hypoxic conditions. EPLC-272H and H1339 cells were treated with 17-AAG (5, 10, 20 nM) or NVP-AUY922 (1, 2 nM)) or vehicle control (0 nM), 30 min later exposed to normoxic (A) or hypoxic (B) conditions for 24 h and, after 5 min of reoxygenation, irradiated with increasing doses of x-rays. Drugs were present until fixation of colonies. The survival fractions were calculated after normalization for cell kill by 17-AAG or NVP-AUY922 alone. The pre-incubation under hypoxia for 24 h had no significant influence on the survival fraction (H1339: 94±3.6%; EPLC-272H: 100±5.3%). Mean values ± SEM of at least 4 independent experiments are shown. Significant differences between vehicle control and cells treated with 20 nM 17-AAG or 2 nM NVP-AUY922 are indicated (*p≤0.05, **p≤0.01). Survival curves were fitted to the linear quadratic model.

Based on the differences in the IC_50_ values of the two drugs, both tumor cell lines were treated for 24 h with 5, 10 and 20 nM 17-AAG, or 1 and 2 nM NVP-AUY922 in a combined treatment with irradiation (0, 2, 4 and 6 Gy). Under normoxic conditions the clonogenic survival curves of EPLC-272H cells revealed no significant differences in radiosensitivity in the presence or absence of 17-AAG or NVP-AUY922 (Fig. 5A, left panel). To our surprise, radiosensitivity of H1339 cells was significantly increased after treatment either with 20 nM 17-AAG (p = 0.003 at 2 Gy; p = 0.007 at 4 Gy) or 2 nM NVP-AUY922 (p = 0.008 at 4 Gy; p = 0.004 at 6 Gy) (Fig. 5A, right panel). As already shown for drug only experiments (Fig. 4), a 10-fold lower concentration of NVP-AUY922 (2 nM) resulted in the same radiosensitization observed for 17-AAG (20 nM) in H1339 cells. The effects that were seen in clonogenic assays with low drug concentrations could be confirmed at high concentrations (Fig. S3).

We have shown that hypoxia differentially regulates HIF-1α levels and its target gene products PAI-1 and CA IX in H1339 and EPLC-272H cells (Figs. 1 and 3). Furthermore, hypoxia can modulate the proteome of tumor cells via the HIF signaling and can cause a more aggressive and radioresistant phenotype [25]. Most importantly, the group of Dewhirst demonstrated that exposure to hypoxia before irradiation influences radiosensitivity and that this effect is HIF-1α dependent [26]. Therefore, we investigated whether 17-AAG- or NVP-AUY922-induced changes in HIF-1α levels determine the radiosensitivity of the tumor cells exposed to hypoxic conditions ([O_2_] = 0.66%) prior to irradiation.

Similar to normoxia, the dose-response curves showed no significant effect of 17-AAG or NVP-AUY922 on the radiosensitivity of hypoxic EPLC-272H cells with a down-regulated HIF-1α expression (Fig. 5B, left panel). In contrast, hypoxic H1339 cells with significantly elevated HIF-1α levels showed a dramatic increase in their radiosensitivity upon treatment with 17-AAG (p = 0.040 at 2 Gy; p = 0.021 at 4 Gy) and NVP-AUY922 (Fig. 5B, right panel).

A thorough analysis of radiobiological parameters confirmed the radiosensitization in H1339 cells treated with 10 and 20 nM 17-AAG or 2 nM NVP-AUY922 under normoxic and hypoxic conditions (Table S2).

**Figure 6:**
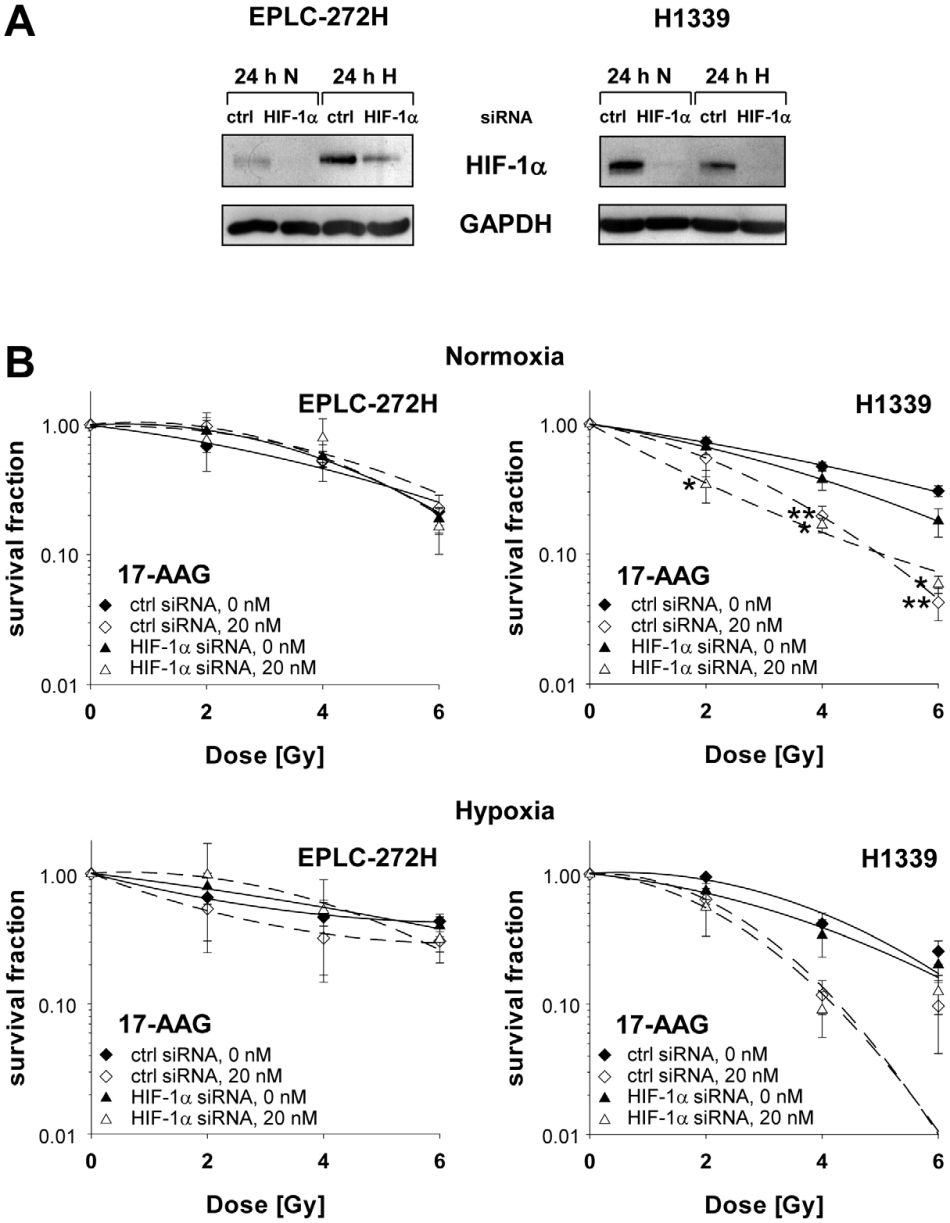
Radiosensitization of H1339 lung cancer cells by 17-AAG is independent of HIF-1α. EPLC-272H and H1339 cells were transfected with non-targeting (ctrl) or HIF-1α siRNA. At 48 h after transfection, cells were treated with vehicle control (0 nM) or 20 nM 17-AAG, 30 min later exposed to normoxic or hypoxic conditions for 24 h and, after 5 min of reoxygenation, irradiated with increasing doses of x-rays. (A) Knock-down of HIF-1α at the time of irradiation is shown by immunoblot. (B) The survival fractions were calculated after normalization for cell kill by 17-AAG alone. 17-AAG was present until fixation of colonies. Mean values ± SEM of 4 independent experiments are shown. Significant differences between vehicle control and cells treated with 20 nM 17-AAG are indicated (*p≤0.05, **p≤0.01). Survival curves were fitted to the linear quadratic model.

To investigate the impact of the Hsp90 inhibitor-induced elevated HIF-1α levels on the radiosensitivity of H1339 cells, HIF-1α expression was down-regulated by a specific siRNA at the time of irradiation (Fig. 6A). EPLC-272H cells in which HIF-1α is induced by hypoxia but not by Hsp90 inhibition were used as a control. In the absence of HIF-1α, radiosensitivity of normoxic and hypoxic EPLC-272H and H1339 cells was not significantly influenced (Fig. 6B). Independently of the HIF-1α status the radiosensitivity of EPLC-272H cells could not be enhanced by 17-AAG treatment (Fig. 6B). However, treatment with 17-AAG (20 nM) significantly sensitized normoxic as well as hypoxic H1339 cells towards irradiation, regardless of their intrinsic HIF-1α levels. These data indicate that Hsp90 inhibition increases radiosensitivity of H1339 lung cancer cells in a HIF-1α-independent manner.

## Discussion

Since hypoxia poses a major problem for the efficacy of radiotherapy in lung cancer patients, the radiosensitizing capacity of a novel (NVP-AUY922) and an established (17-AAG) Hsp90 inhibitor was analyzed in human lung carcinoma cell lines under normoxic and hypoxic conditions and at clinically relevant, low concentrations. In previous studies these effects were studied only under normoxia and at high inhibitor concentrations [10], [27]–[30].

In general, Hsp90 inhibitor-induced effects on the regulation of hypoxia inducible genes are still a matter of debate. Ibrahim et al. [31] described a 17-AAG-induced up-regulation of HIF-1α at low (5–30 nM) and a down-regulation at high inhibitor concentrations (1000–3000 nM). An oxygen- and VHL/PHD-independent ubiquitination and proteasomal degradation of HIF-1α was observed after incubation of tumor cells with geldanamycin [32]–[34] and the small molecule inhibitor NVP-AUY922 has been shown to reduce HIF-1α levels in human tumor xenografts [35]. In line with these findings, upon treatment with 17-AAG and NVP-AUY922, basal as well as hypoxia-induced HIF-1α levels were found to be decreased in normoxic and hypoxic EPLC-272H cells at any tested concentration. In contrast, H1339 tumor cells show an up-regulated HIF-1α expression upon Hsp90 inhibition, irrespectively of the inhibitor concentration and oxygenation status.

A dual role has been shown for Hsp70, on the one hand Hsp70 can protect HIF-1α from Hsp90 inhibitor induced degradation [36] and on the other hand Hsp70 has been found to contribute to the Hsp90 inhibitor induced degradation of HIF-1α [16]. However, no significant differences were found in the Hsp70 induction upon Hsp90 inhibition in H1339 and EPLC-272H cells, with differential HIF-1α expression. Therefore, it was assumed that genetic alterations in oncogenes and/or tumor suppressor genes might be involved in the tumor cell type-specific regulation of HIF-1α. Receptor of activated protein C kinase (RACK1) competes with Hsp90 for binding to the Per/ARNT/Sim (PAS) -A domain of HIF-1α and is required for Hsp90 inhibitor-induced degradation of HIF-1α [17]. However, H1339 and EPLC-272H tumor cells express similar amounts of RACK1 and also sequence analysis did not reveal any mutations in the PAS-A domain of HIF-1α in H1339 cells.

Cullin 5 E3 ubiquitin ligase (Cul5) and the Copper Metabolism MURR1 Domain containing 1 protein (COMMD1) have recently been reported to play key roles in the Hsp90 inhibitor-induced degradation of HIF-1α [16]–[18]. Most notably, COMMD1 and Cul5 expression was found to be decreased or lost in a number of human tumors [37], [38]. However, with respect to the expression pattern of these proteins upon Hsp90 inhibition, no differences were detectable in H1339 and EPLC-272H tumor cells.

Apart from HIF-1α, other HIF-α-subunits interact with Hsp90 [5]. In HIF-2α stably transfected Chinese hamster ovary (CHO) cells, a treatment with geldanamycin caused a down-regulation of HIF-2α [39]. We found that basal and hypoxia-induced naturally occurring HIF-2α levels in EPLC-272H and H1339 lung cancer cells remained unaltered following incubation with 17-AAG and NVP-AUY922. The different results might be explained by the fact that CHO cells are stably transfected with HIF-2α, whereas the human lung cancer cell lines endogenously express HIF-2α.

The lung cancer cell lines EPLC-272H and H1339 were chosen because they differ in their basal HIF-1α levels but show comparable intrinsic resistance towards irradiation or drug alone. Upon Hsp90 inhibition, HIF-1α levels were further elevated in H1339 cells, whereas, normoxic and hypoxic EPLC-272H cells reacted as expected with a reduction of HIF-1α.

Since high HIF-1 activity has been linked to radioresistance [11], [12], [40]–[42], an Hsp90 inhibitor driven radiosensitization has been expected for EPLC-272H cells with decreased HIF-1α levels. To our surprise, radiosensitization was only observed in H1339 cells expressing high HIF-1α levels upon Hsp90 inhibition under normoxic and hypoxic conditions. An intrinsic tolerance of EPLC-272H cells to Hsp90 inhibitors could be excluded since both lung cancer cell lines showed a comparable up-regulation of Hsp70 – a surrogate marker for Hsp90 inhibition - and a similar sensitivity towards drug alone.

However, dual effects of HIF-1α in regulating tumor cell survival have been described [26]. On the one hand, HIF-1α can exert pro-survival functions, e.g., by the inhibition of apoptosis, on the other hand, HIF-1α can increase apoptosis, enhance glycolysis and induce mitosis in tumor cells, all factors leading to increased radiosensitivity [43]–[46]. Therefore, the balance of these opposing effects may determine whether or not HIF-1α has an effect on radiosensitivity.

In summary, our data reveal that radiosensitization of tumor cells by Hsp90 inhibitors is independent of basal and Hsp90 inhibitor-induced HIF-1α levels.

## Materials and Methods

### Reagents

10 mM stock solutions of 17-AAG (Sigma, Taufkirchen, Germany) and NVP-AUY922 (Novartis, Basel, Switzerland) were prepared in 100% DMSO and aliquots stored at −20°C. Further dilutions were performed in PBS. A vehicle control with the respective amount of DMSO diluted in PBS (0.00001%–0.01% DMSO ≙ 1 nM–1000 nM NVP-AUY922 or 17-AAG) was tested in all experiments to exclude an effect of DMSO itself.

### Cells and cell culture

Human lung cancer cell lines, H1339 (SCLC) and EPLC-272H (NSCLC) (kindly provided by Prof. Rudolf Huber, Department of Pneumology, University of Munich, Munich, Germany) were cultured in RPMI 1640 (Invitrogen, San Diego, CA) supplemented with 10% v/v heat-inactivated FCS (PAA, Pasching, Austria), 100 IU/ml penicillin, 100 µg/ml streptomycin, 2 mM L-glutamine, and 1 mM sodium pyruvate (Life Technologies, Eggenstein, Germany). Cells were routinely checked and determined as negative for mycoplasma contamination.

### Hypoxia

Cells were incubated for various time periods (2, 8, 16 and 24 h) under hypoxic conditions ([O_2_] = 0.66%) at 37°C. Hypoxic conditions were achieved as described previously [47].

### ELISA and Western blot analysis

Attached cells were lysed in TBST buffer (1% Triton X-100 in TBS, 1 mM PMSF, protease inhibitor cocktail) as described previously [48]. Protein content in the cell lysates was determined using the BCA™ Protein Assay Kit (Pierce, Rockford, IL).

HIF-1α concentrations in cell lysates (R & D Systems, Minneapolis, MN) were determined by ELISA according to the manufacturer's instructions. HIF-1α concentrations were calculated relative to the total protein content of each sample. For the calculation of relative levels, the untreated control was set at 100%.

For Western Blot analysis, proteins were separated by SDS-PAGE under reducing conditions as previously described [49], blotted onto PVDF membranes and detected with monoclonal antibodies directed against HIF-1α (R & D Systems, Minneapolis, MN), Hsp70 (SPA-810, Assay Designs, Ann Arbor, MI), RACK1 (BD, NJ, USA), COMMD1 (Abnova, Taipei City, Taiwan), GAPDH (Sigma-Aldrich, Taufkirchen, Germany), β-actin (Sigma-Aldrich, Taufkirchen, Germany) and polyclonal antibodies directed against HIF-2α (abcam, Cambridge, UK), AKT (Cell Signaling, MA, USA), Cul5 (Millipore, MA; USA), CA IX (Lifespan Biosciences, Seattle, WA) and PAI-1 (kindly provided by Prof. Sweep, Department of Chemical Endocrinology, Radboud University Nijmegen Medical Center, Nijmegen, Netherlands). Bound antibodies were visualized using horseradish peroxidise-conjugated secondary antibodies (Promega, Madison, WI) and a chemiluminescence developing kit (ECL, Amersham Biosciences, Buckinghamshire, UK). The protein bands were quantified by densitometry using ImageJ.

### Transfection with siRNA

Cells were transfected with 5 nM siRNA duplexes (Qiagen, Hilden, Germany) to either a non-targeting sequence (allstars negative control), to HIF-1α (AGGAAGAACTATGAACATAAA), to HIF-2α (CCCGGATAGACTTATTGCCAA) or to both HIF-1α and HIF-2α using the HiPerfect Transfection Reagent (Qiagen, Hilden, Germany).

### Clonogenic cell survival assay and irradiation

Tumor cells were seeded into 12-well plates. Cells were treated with 17-AAG or NVP-AUY922, exposed to hypoxia or normoxia and/or irradiated. Cells were irradiated with the indicated doses using the RS225A irradiation device (Gulmay Medical Ltd., UK) at a dose rate of 1 Gy/min (70 keV). Irradiations were performed under atmospheric conditions ([O_2_] = 21%). On day 7–10 after seeding, colonies were fixed in methanol, stained with crystal violet and counted. Survival curves were fitted to the linear quadratic model using Sigmaplot (Systat Software Inc, San Jose, CA).

### Statistics

Statistical analysis was performed using SPSS 18.0.2 software. The Student's t-test was used to evaluate significant differences (*p≤0.05, **p≤0.01, ***p≤0.001).

## Supporting Information

Figure S1 **Hypoxia induced G_1_-arrest in H1339 cells.** Following normoxia (24 h N) or hypoxia (24 h H) for 24 h, cells were harvested. After washing in PBS and fixation (70% ethanol over night; −20°C), cells were incubated in PI staining solution (PBS containing 0.1% Triton X-100, 0.2 mg/ml RNase A, 0.02 mg/ml PI) for 1 h at room temperature and analyzed on a FACSCalibur flow cytometer (BD Biosciences, San Jose, CA, USA). Cell cycle distribution was determined using ModFit LT (Verity Software House, Topsham, ME). Data represent mean values ± SEM of three independent experiments. After hypoxia G_1_-phase was significantly (*** *p*≤0.001) increased and S-phase significantly (*** *p*≤0.001) reduced.
(TIFF)

Figure S2 **GAPDH and β-actin are comparable loading controls in normoxic and hypoxic EPLC-272H and H1339 tumor cells.** Following treatment (30 min) with 0, 100 and 1000 nM 17-AAG, EPLC-272H and H1339 cells were exposed to normoxia (24 h N) or hypoxia (24 h H) for 24 h. The expression of GAPDH and ß-actin was comparable in untreated and 17-AAG-treated cells under normoxic and hypoxic conditions. Similar results were obtained for NVP-AUY922 (data not shown).
(TIF)

Figure S3 **Radiosensitivity of EPLC-272H and H1339 cells after treatment with high concentrations of 17-AAG.** 24 h after treatment of EPLC-272H and H1339 tumor cells with 100 nM 17-AAG or vehicle control (0 nM), cells were irradiated with increasing doses of x-rays. 17-AAG was removed 1 h after irradiation. The survival fractions were calculated after normalization for cell kill by 17-AAG alone. Data represent mean values ± SEM of at least 3 independent experiments. Significant differences between vehicle control and cells treated with 100 nM 17-AAG are indicated (*p≤0.05, **p≤0.01). Survival curves were fitted to the linear quadratic model.
(TIFF)

Table S1 **Percentage of viable cells treated for 24 h with 17-AAG or NVP-AUY922.** Following a 24 h treatment with 17-AAG or NVP-AUY922, cells were washed and stained with propidium iodide (PI) for 1 min. Viable cells (PI negative) were analyzed on a FACSCalibur flow cytometer (BD Biosciences, San Jose, CA, USA). Mean values ± SEM of four independent experiments are shown.
(DOC)

Table S2 **Summary of radiobiological parameters calculated from** **Fig. 5** **.** SF_2_, survival fraction at 2 Gy. D_50_, dose to reduce survival fraction to 50%. Sensitizing enhancement ratio (SER) = D_50_ (irradiation)/D_50_ (irradiation and drug). A SER greater than 1.20 is indicative for radiosensitization (indicated in bold). Mean values ± SEM of at least four independent experiments are shown.
(DOC)
