# Supplementary figures and images for: Radiosensitization of Normoxic and Hypoxic H1339 Lung Tumor Cells by Heat Shock Protein 90 Inhibition Is Independent of Hypoxia Inducible Factor-1α
Source: PLoS One. 2012 Feb 7;7(2):e31110. doi: 10.1371/journal.pone.0031110 (PMC3274537; doi:10.1371/journal.pone.0031110)

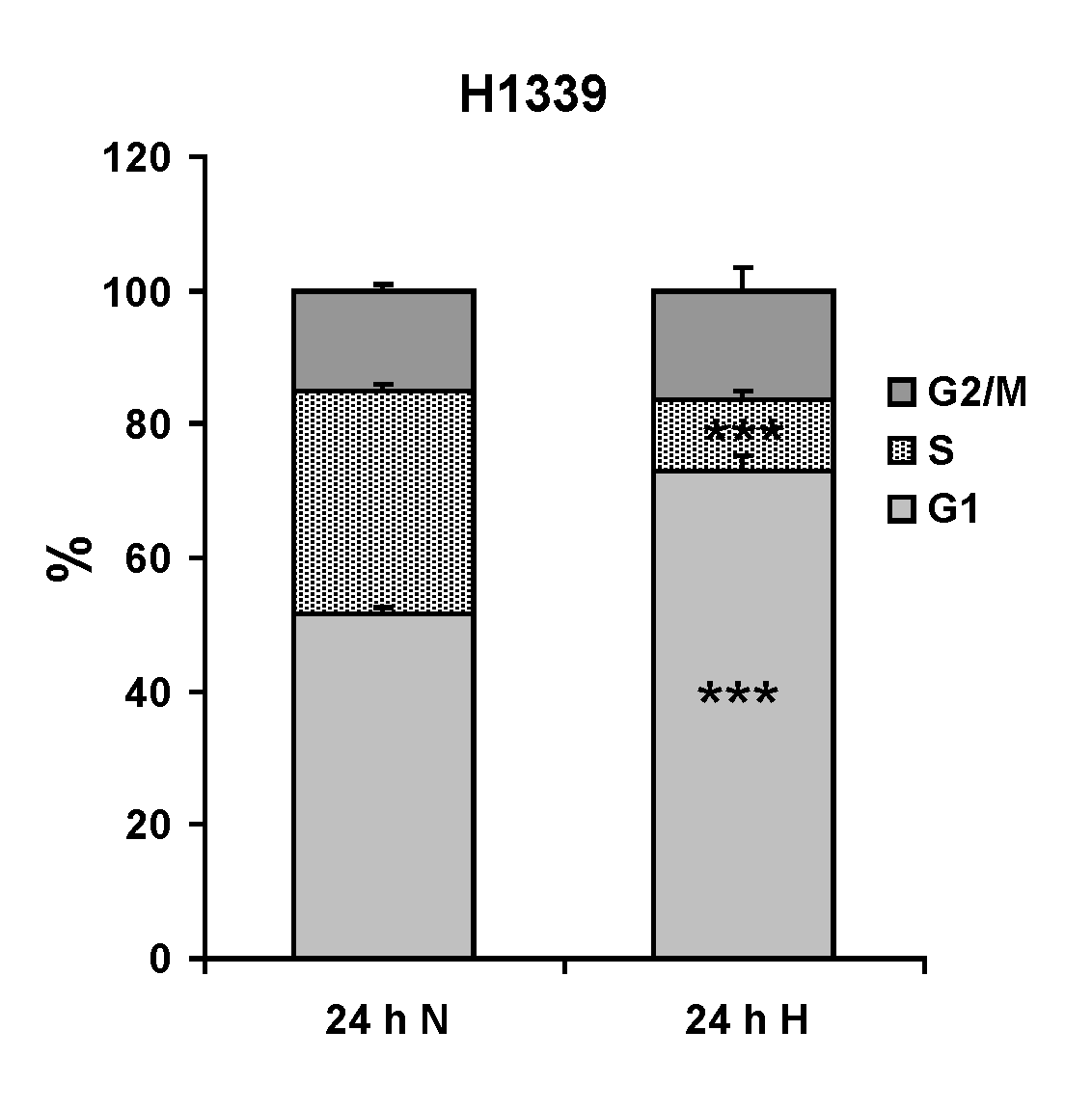

Supplement: Figure S1 — Hypoxia induced G1-arrest in H1339 cells. Following normoxia (24 h N) or hypoxia (24 h H) for 24 h, cells were harvested. After washing in PBS and fixation (70% ethanol over night; −20°C), cells were incubated in PI staining solution (PBS containing 0.1% Triton X-100, 0.2 mg/ml RNase A, 0.02 mg/ml PI) for 1 h at room temperature and analyzed on a FACSCalibur flow cytometer (BD Biosciences, San Jose, CA, USA). Cell cycle distribution was determined using ModFit LT (Verity Software House, Topsham, ME). Data represent mean values ± SEM of three independent experiments. After hypoxia G1-phase was significantly (*** p≤0.001) increased and S-phase significantly (*** p≤0.001) reduced. (TIFF) [file pone.0031110.s001.tiff]

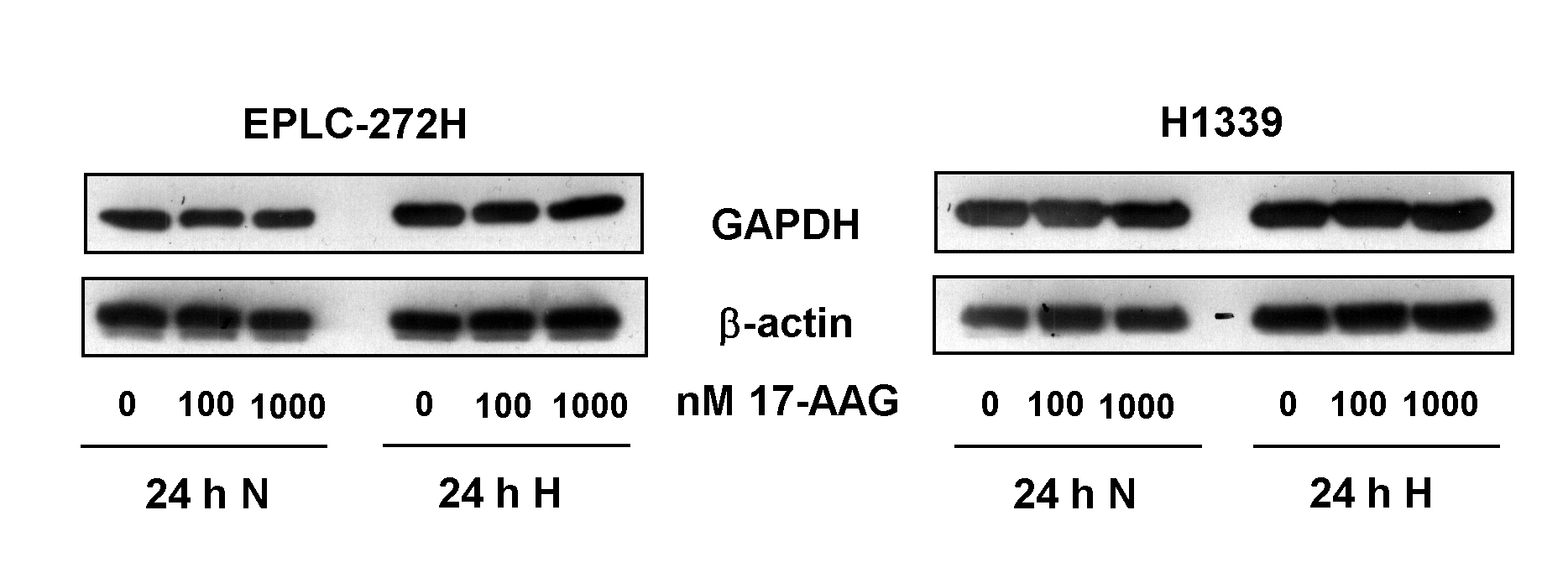

Supplement: Figure S2 — GAPDH and β-actin are comparable loading controls in normoxic and hypoxic EPLC-272H and H1339 tumor cells. Following treatment (30 min) with 0, 100 and 1000 nM 17-AAG, EPLC-272H and H1339 cells were exposed to normoxia (24 h N) or hypoxia (24 h H) for 24 h. The expression of GAPDH and ß-actin was comparable in untreated and 17-AAG-treated cells under normoxic and hypoxic conditions. Similar results were obtained for NVP-AUY922 (data not shown). (TIF) [file pone.0031110.s002.tif]

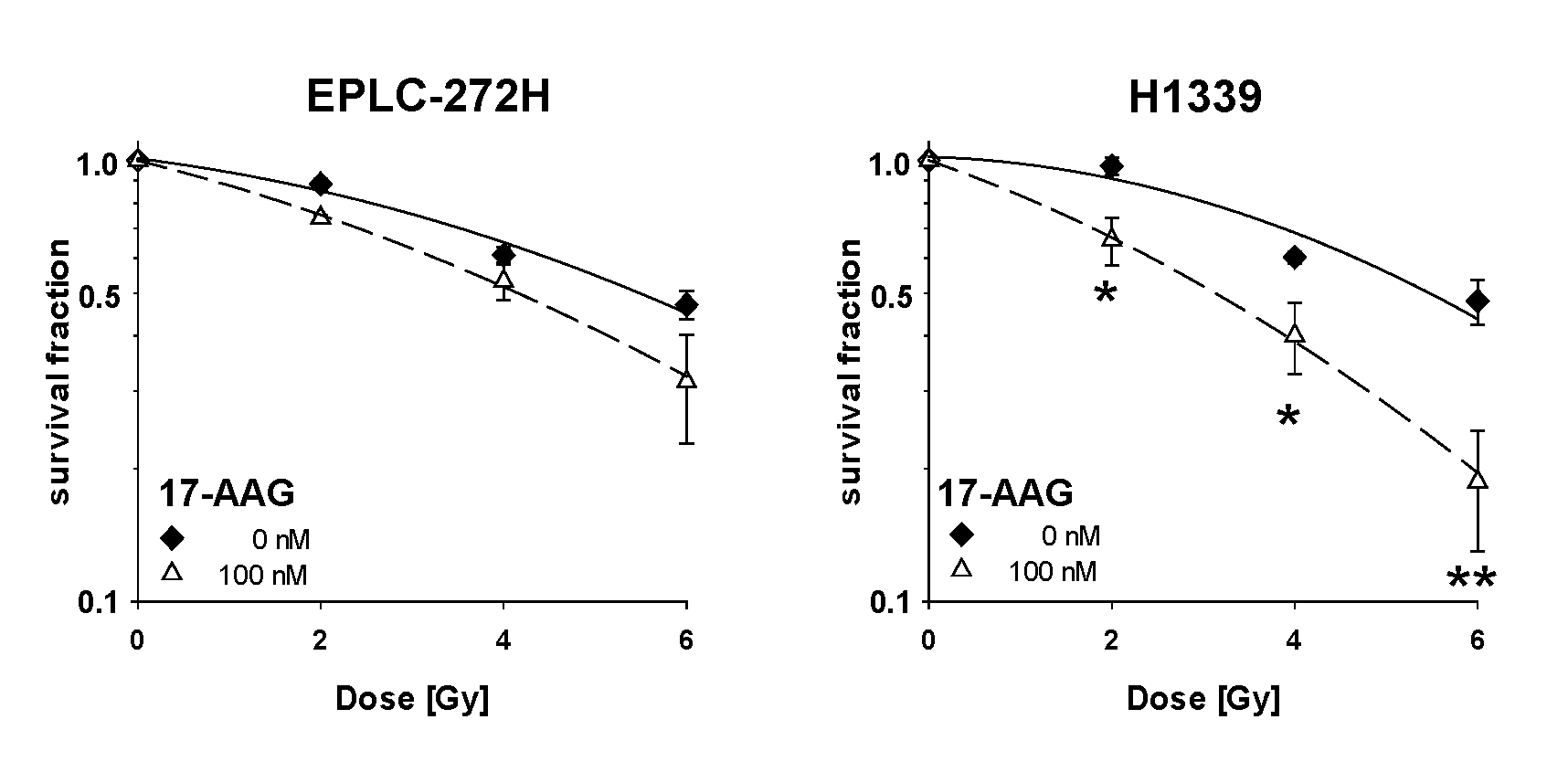

Supplement: Figure S3 — Radiosensitivity of EPLC-272H and H1339 cells after treatment with high concentrations of 17-AAG. 24 h after treatment of EPLC-272H and H1339 tumor cells with 100 nM 17-AAG or vehicle control (0 nM), cells were irradiated with increasing doses of x-rays. 17-AAG was removed 1 h after irradiation. The survival fractions were calculated after normalization for cell kill by 17-AAG alone. Data represent mean values ± SEM of at least 3 independent experiments. Significant differences between vehicle control and cells treated with 100 nM 17-AAG are indicated (*p≤0.05, **p≤0.01). Survival curves were fitted to the linear quadratic model. (TIFF) [file pone.0031110.s003.tiff]
