# Supplementary material for: Radiosensitization of Normoxic and Hypoxic H1339 Lung Tumor Cells by Heat Shock Protein 90 Inhibition Is Independent of Hypoxia Inducible Factor-1α
Source: PLoS One. 2012 Feb 7;7(2):e31110. doi: 10.1371/journal.pone.0031110 (PMC3274537; doi:10.1371/journal.pone.0031110)
Supplement: Table S1 — Percentage of viable cells treated for 24 h with 17-AAG or NVP-AUY922. Following a 24 h treatment with 17-AAG or NVP-AUY922, cells were washed and stained with propidium iodide (PI) for 1 min. Viable cells (PI negative) were analyzed on a FACSCalibur flow cytometer (BD Biosciences, San Jose, CA, USA). Mean values ± SEM of four independent experiments are shown. (DOC) [file pone.0031110.s004.doc]

**Table S1: Percentage of viable cells treated for 24 h with 17-AAG or NVP-AUY922.**

|  |  | 17-AAG | | NVP-AUY922 | |
| --- | --- | --- | --- | --- | --- |
|  | 0 nM | 100 nM | 1000 nM | 100 nM | 1000 nM |
| H1339 | 90.5 ± 1.3 | 89.7 ± 1.3 | 90.3 ± 0.8 | 90.6 ± 0.9 | 89.5 ± 1.0 |
| EPLC-272H | 94.5 ± 0.7 | 92.5 ± 0.8 | 93.7 ± 0.6 | 91.2 ± 1.1 | 92.5 ± 1.0 |

Following a 24 h treatment with 17-AAG or NVP-AUY922, cells were washed and stained with propidium iodide (PI) for 1 min. Viable cells (PI negative) were analyzed on a FACSCalibur flow cytometer (BD Biosciences, San Jose, CA, USA). Mean values ± SEM of four independent experiments are shown.
