# Supplementary material for: Radiosensitization of Normoxic and Hypoxic H1339 Lung Tumor Cells by Heat Shock Protein 90 Inhibition Is Independent of Hypoxia Inducible Factor-1α
Source: PLoS One. 2012 Feb 7;7(2):e31110. doi: 10.1371/journal.pone.0031110 (PMC3274537; doi:10.1371/journal.pone.0031110)
Supplement: Table S2 — Summary of radiobiological parameters calculated from Fig. 5 . SF2, survival fraction at 2 Gy. D50, dose to reduce survival fraction to 50%. Sensitizing enhancement ratio (SER) = D50 (irradiation)/D50 (irradiation and drug). A SER greater than 1.20 is indicative for radiosensitization (indicated in bold). Mean values ± SEM of at least four independent experiments are shown. (DOC) [file pone.0031110.s005.doc]

**Table S2: Summary of radiobiological parameters calculated from Fig. 5.**

|  | **EPLC-272H** | | | **H1339** | | |
| --- | --- | --- | --- | --- | --- | --- |
|  | **SF2** | **D50** | **SER** | **SF2** | **D50** | **SER** |
| **Normoxia** |  |  |  |  |  |  |
| 0 nM 17-AAG | 0.64 ± 0.10 | 3.82 | 1.00 | 0.76 ± 0.06 | 3.79 | 1.00 |
| 5 nM 17-AAG | 0.72 ± 0.11 | 3.94 | 0.97 | 0.71 ± 0.04 | 3.28 | 1.15 |
| 10 nM 17-AAG | 0.70 ± 0.05 | 3.48 | 1.10 | 0.52 ± 0.13 | 2.27 | **1.67** |
| 20 nM 17-AAG | 0.80 ± 0.08 | 5.20 | 0.73 | 0.44 ± 0.04 | 1.73 | **2.18** |
| 0 nM NVP-AUY922 | 0.67 ± 0.02 | 3.73 | 1.00 | 0.75 ± 0.04 | 4.04 | 1.00 |
| 1 nM NVP-AUY922 | 0.77 ± 0.02 | 4.10 | 0.91 | 0.71 ± 0.07 | 3.60 | 1.12 |
| 2 nM NVP-AUY922 | 0.65 ± 0.10 | 3.72 | 1.00 | 0.58 ± 0.07 | 2.67 | **1.51** |
| **Hypoxia** |  |  |  |  |  |  |
| 0 nM 17-AAG | 0.68 ± 0.11 | 4.05 | 1.00 | 0.66 ± 0.04 | 3.20 | 1.00 |
| 5 nM 17-AAG | 0.60 ± 0.12 | 3.50 | 1.16 | 0.70 ± 0.10 | 3.11 | 1.03 |
| 10 nM 17-AAG | 0.64 ± 0.16 | 3.67 | 1.10 | 0.56 ± 0.10 | 2.50 | **1.28** |
| 20 nM 17-AAG | 0.64 ± 0.20 | 4.97 | 0.81 | 0.43 ± 0.09 | 1.63 | **1.97** |
| 0 nM NVP-AUY922 | 0.69 ± 0.05 | 3.81 | 1.00 | 0.68 ± 0.04 | 3.36 | 1.00 |
| 1 nM NVP-AUY922 | 0.80 ± 0.05 | 4.05 | 0.94 | 0.73 ± 0.08 | 3.37 | 1.00 |
| 2 nM NVP-AUY922 | 0.77 ± 0.13 | 5.16 | 0.74 | 0.49 ± 0.09 | 2.13 | **1.58** |

SF_2_, survival fraction at 2 Gy. D_50_, dose to reduce survival fraction to 50%. Sensitizing enhancement ratio (SER) = D_50_ (irradiation)/D_50_ (irradiation and drug). A SER greater than 1.20 is indicative for radiosensitization (indicated in bold). Mean values ± SEM of at least four independent experiments are shown.
